# Trade, investment and public health: compiling the evidence, assembling the arguments

**DOI:** 10.1186/s12992-018-0425-y

**Published:** 2019-01-03

**Authors:** Ronald Labonté

**Affiliations:** University of Ottawa, Ottawa, Canada

## Abstract

Trade has long been an axiomatic characteristic of globalization, although international rules governing trade are of more recent vintage. Notably in the post-World War II period, an ever increasing number of countries began negotiating treaties to reduce, first, tariff barriers and, later, non-tariff barriers (government measures of any sort) that could impede the cross-border flow of goods. The rationale, in part, was that countries that became more entwined economically would be less likely to go to war with each other. It wouldn’t be in their own economic interests to do so, or at least that of the firms based within their borders but engaged in transnational trade and dependent upon global supply chains. At first primarily an undertaking of developed (high-income) countries, developing (low and middle-income countries) slowly enjoined in what, in 1995, became the World Trade Organization. The WTO locked in scheduled declines in tariffs (border taxes), albeit with lesser obligations on developing country members (a problematic nomenclature given the vast geographic, economic, and development differences between such countries, but which nonetheless persists within the WTO). Importantly, a slew of new agreements that coincided with the establishment of the WTO also sought to liberalize trade in services (not just goods) (The General Agreement on Trade in Services), create new rules for agricultural trade (Agreement on Agriculture), expand intellectual property rights protections (The Agreement on Trade-Related Aspects of Intellectual Property Rights), limit trade-distorting government subsidies (Agreement on Subsidies and Countervailing Measures), and ensure that government food, health, or environmental regulations would not pose an unnecessary barrier to trade (the Technical Barriers to Trade and the Sanitary and Phytosanitary Measures Agreements). Outside of the WTO system, bilateral or regional investment treaties granting special rights to foreign investors to sue governments for actions perceived to affect the value of their investment (such as direct expropriation or passage of new laws and regulations considered ‘tantamount to expropriation’) similarly exploded in number, dispute frequency, and the size of monetary claims. The breadth and depth of these post-1995 Agreements meant that few areas of general public health concern are potentially untouched.

## Collected articles from *Globalization and Health* (2006–2018)

Trade has long been an axiomatic characteristic of globalization, although international rules governing trade are of more recent vintage. Notably in the post-World War II period, an ever increasing number of countries began negotiating treaties to reduce, first, tariff barriers and, later, non-tariff barriers (government measures of any sort) that could impede the cross-border flow of goods. The rationale, in part, was that countries that became more entwined economically would be less likely to go to war with each other. It wouldn’t be in their own economic interests to do so, or at least that of the firms based within their borders but engaged in transnational trade and dependent upon global supply chains. At first primarily an undertaking of developed (high-income) countries, developing (low and middle-income countries) slowly enjoined in what, in 1995, became the World Trade Organization. The WTO locked in scheduled declines in tariffs (border taxes), albeit with lesser obligations on developing country members (a problematic nomenclature given the vast geographic, economic, and development differences between such countries, but which nonetheless persists within the WTO). Importantly, a slew of new agreements that coincided with the establishment of the WTO also sought to liberalize trade in services (not just goods) (The General Agreement on Trade in Services), create new rules for agricultural trade (Agreement on Agriculture), expand intellectual property rights protections (The Agreement on Trade-Related Aspects of Intellectual Property Rights), limit trade-distorting government subsidies (Agreement on Subsidies and Countervailing Measures), and ensure that government food, health, or environmental regulations would not pose an unnecessary barrier to trade (the Technical Barriers to Trade and the Sanitary and Phytosanitary Measures Agreements). Outside of the WTO system, bilateral or regional investment treaties granting special rights to foreign investors to sue governments for actions perceived to affect the value of their investment (such as direct expropriation or passage of new laws and regulations considered ‘tantamount to expropriation’) similarly exploded in number, dispute frequency, and the size of monetary claims. The breadth and depth of these post-1995 Agreements meant that few areas of general public health concern are potentially untouched.

There is nothing intrinsically unhealthy about international trade. Whether trade or foreign investment lead to health-enhancing or health-damaging outcomes related to social, economic, or regulatory changes depends very much on the specific and binding rules of particular agreements. Food trade can increase the availability, and even the affordability, of healthy foods but it can also flood markets with obesogenic (and more readily affordable) food products. Health services trade could improve the quality of care in many countries, but it could also increase privatization in such services and crowd out access for low-income populations. Intellectual property rights can incentivize new drug discoveries but price essential medicines beyond the affordability means of the poor or their governments. At an aggregate level, global trade can increase economic growth with potential trickle-down income growth and related health benefits, but not all countries will benefit equitably (if at all) and benefits within countries may be skewed in favour of some populations, but not others. To the extent that trade-related economic growth increases negative environmental externalities (such as climate change and resource depletion), it contributes indirectly to what are now increasingly central public health concerns. Trade rules could be used to further compliance with international environmental law, and to reduce barriers to the diffusion of ‘green technologies’; but they can also be used (and have been) to challenge countries’ subsidies or supports for the production and export of such technologies.

This ambivalent or dialectical relationship between trade and health led to a slowly growing research scholarship, some of it published in this journal. Indeed, the inaugural issue of this journal featured its first article on trade and health, a critical assessment of the potential impacts of a new Australian and US Free Trade Agreement (AUSFTA) of extended intellectual property rights (IPRs) on “equitable and affordable access to essential medicines” [1]. (p15) Concerns over the impact of IPRs on drug costs have featured prominently in research on the trade/health nexus, including several more in subsequent years in *Globalization and Health*, and represent a public health particularism that focuses on a single pathway by which trade or investment treaties might affect a specific health outcome or determinant (in this instance, access to medicines). The detailing in such studies offers depth and specificity to the trade/health relationship, but at the cost of less breadth given to the pervasiveness of potential trade-related health impacts. Other trade-related health issues, however, have also garnered critical attention and study, many with respect to tobacco, food and dietary transitions, and non-communicable diseases; as well as concerns with more generic trade-related impacts on health services and labour markets. A few studies have undertaken health impact assessments of ‘new generation’ regional free trade agreements (FTAs) that arose in the wake of stalled negotiations under the multilateral WTO system, expanding upon the breadth of health effects associated with both trade and investment treaty provisions.

In this special collection we draw together 17 trade and health articles that have appeared in this journal over the past 12 years, organized thematically (see Overview). The changeable landscape of trade and investment treaties, to say nothing of ongoing treaty negotiations and re-negotiations, means that some of these contributions are not as current as yesterday’s news; a certain interpretative caution is therefore issued to readers. At the same time, our compilation of articles is based, in part, on them offering still useful commentaries, perspectives, and research findings on a global policy domain that is often complex and almost always contentious. In this overview, we also supplement these notable contributions to the trade and health nexus by referencing other studies, often by the same authors, that interrogate the same or similar questions.

**Table:** 

1. Trade, investment and non-communicable diseases	
• Framing international trade and chronic disease.	
• Trade and investment liberalization and Asia’s noncommunicable disease epidemic: a synthesis of data and existing literature	
2. Elaborating the evidence base	
• Uneven dietary development: linking the policies and processes of globalization with the nutrition transition, obesity and diet-related chronic diseases	
• Overweight in the Pacific: links between foreign dependence, global food trade, and obesity in the Federated States of Micronesia	
• The implications of trade liberalization for diet and health: a case study from Central America	
3. The natural experiments	
• The role of trade and investment liberalization in the sugar-sweetened carbonated beverages market: a natural experiment contrasting Vietnam and the Philippines	
• Trade and Investment liberalization, food systems change and ultra-processed food consumption: a natural experiment contrasting the soft drinks markets of Peru and Bolivia	
4. The health impact assessment studies	
• A new generation of trade policy: potential risks to diet-related health from the trans pacific partnership agreement	
• The Trans-Pacific Partnership Agreement and health: few gains, some losses, many risks	
5. Access to essential medicines vs. drug patents and profits	
• TRIPS, the Doha declaration and paragraph 6 decision: what are the remaining steps for protecting access to medicines?	
• Canada's implementation of the Paragraph 6 Decision: is it sustainable public policy?	
• Canada and access to medicines in developing countries: intellectual property rights first.	
6. Broadening the trade/health Nexus	
• India-EU relations in health services: prospects and challenges	
• Trade liberalization, social policies and health: an empirical case study.	
• Improving regulatory capacity to manage risks associated with trade agreements	
7. Forward research directions	
• The health impact of trade and investment agreements: a quantitative systematic review and network co-citation analysis	
• Analyzing the impacts of global trade and investment on non-communicable diseases and risk factors: a critical review of methodological approaches used in quantitative analyses.	

### Overview

### Trade, investment and non-communicable diseases

Several of the articles published in *Globalization and Health* examine the effects of trade and investment treaties on non-communicable diseases (NCDs). One of the earlier contributions takes a broad-brushed approach, reviewing existing studies on trade-related impacts on NCD prevalence, notably in low- and middle-income countries (LMICs) that represent new markets for the three ‘unhealthy commodities’ that are this article’s focus: obesogenic (ultra-processed) foods, tobacco, and alcohol [2]. After reviewing key enforceable principles that govern all trade rules (e.g. national treatment and most favoured nation),^1^ the authors construct a generic framework identifying how trade rules (and trade that falls outside of any treaty arrangements) affect the global diffusion of unhealthy commodities and, eventually, NCD outcomes (both positive and negative). Its discussion of trade-related food pathways acknowledges that urbanization in LMICs (itself associated with increased trade flows) is another potential explanatory factor in increasing NCD risk (through more sedentary lifestyles), but one that falls outside the article’s review parameters. A later study by one of the authors [3], using trend analyses and structural equation modelling to differentiate the contributions of urbanization and trade/investment on NCD in Sub-Saharan Africa (SSA), found that “global economic integration (trade and investment), beyond the pure generation of wealth (GDP), is linked to intermediate (overweight and obesity) and distal (CVD death) health outcomes,” and explained more of the variance in outcomes than did urbanization prevalence [3] (p299). The article’s synthesis of studies on trade and tobacco was more definitive, finding consistent correlations between liberalized trade and investment in tobacco products, with increases in availability and pricing competition leading to increased consumption levels. Tobacco control policies have subsequently figured prominently in trade and investment disputes, culminating in the failed attempts by tobacco transnational companies and some tobacco-producing countries to ‘chill’ new tobacco control measures. Findings on alcohol trade and risks due to excess consumption were more ambivalent, although the article notes how government policies to regulate consumption (in this case a prima facie non-discriminatory excise tax based on alcohol content) could still violate non-discrimination rules under WTO agreements by setting large tax increases on content levels found in imported, but not domestically produced, spirits.

Less equivocal findings are provided in a later study focusing on trade and investment liberalization and the rising epidemic of NCDs in Asia [4]. Similar to the article described above, this study brought together data on per capita consumption trends in a number of Asian countries for three proximal determinants of NCD risk: tobacco, alcohol, and ultra-processed foods. It linked these trends to a semi-structured literature review assessing probable trade and investment treaty measures that underpinned the observed patterns. Over the study years (1999–2017) tobacco consumption trends were mixed, with declines in many of the studied countries (Singapore, Japan, Korea, and Malaysia), relatively flat in India and the Philippines, but on an increasing trajectory in China, Indonesia and, dramatically so, in Vietnam, a country which had only recently acceded to the WTO or opened itself to foreign direct investment (FDI). Alcohol consumption rose in all study countries, except for Japan. All countries experienced increases in processed food and soft drink consumption. The literature review posited five pathways by which trade and investment liberalization could explain such trends: reductions in tariff and non-tariff barriers; services trade affecting manufacturing and advertising; barriers to FDI incentivizing domestic production of such commodities; increased trade compliance costs reducing public funding for public health prevention programs; and, more broadly, the existence of enforceable trade and investment rules impinging upon the regulatory sovereignty of states.

NCD prevention and mitigation continues to drive much global health policy discourse, at the WHO and in other multilateral governance forums. United Nations ‘high-level meetings’ on NCDs (by 2018, three such intergovernmental meetings had been convened) and a ‘high-level commission’ that reported in 2018 continue to press for implementation of policies to reduce risk factors. Despite the concern in the public health literature such as these two journal contributions, only limited attention has been given to trade and investment treaty provisions as potential drivers of increased NCD morbidity and mortality. More emphasis in global NCD policy discourse is given to ‘lifestyle’ factors (unhealthy behavioural choices) than to the ‘commercial determinants’ of poor health embedded in the market expansion practices of transnational food, tobacco, and alcohol companies, an expansion aided, in part, by trade and investment liberalization treaties.

### Elaborating the evidence base

Some of the articles in this collection emphasize trade or investment treaty provisions for how they might influence health outcomes. Others address trade and investment more by reference to aggregate flows, rather than treaty provisions per se, and how these flows are associated with changes in risk factors for health. A key cynosure has been food, specifically how globalization policies and processes are linked to a ‘nutrition transition’ in which “the consumption of foods high in fats and sweeteners is increasing throughout the developing world” [5] (p4). An early article by Corinna Hawkes (2006) locates the shifts in such consumption within a cluster of interrelated globalization policies and processes, including the growth in transnational food corporations, vertical integration in agricultural supply chains, and new trade and investment liberalization measures that, together, have created a global agri-food system dominated by a handful of powerful corporate actors. As have other health and globalization researchers (e.g. see [6–8]) and heterodox development economists (e.g. see [9]), she identifies the start of this transition in the 1980s and 1990s structural adjustment programs of the World Bank and the International Monetary Fund (which required developing countries receiving adjustment loans to undertake considerable liberalization measures), continuing with the birth of the WTO in 1995 (notably its Agreement on Agriculture), and flanked by the rise in regional FTAs. She illustrates her arguments with case studies of the post-liberalization growth in vegetable oil consumption (citing data from Brazil, China, and India), a major health implication being the rise in hydrogenated *trans* fats; the role of foreign direct investment (FDI) in enabling food processing and expansion of food retailing within developing country borders with reference to the North American Free Trade Agreement (NAFTA) and the surge in obesogenic food consumption in Mexico; and the globalization of food marketing with its impacts on snack-food advertising and consumption in Thailand. Hawkes does not explore the trade agreement specifics of the Thai example, although a later article in this collection (to which we will return) notes how Thailand’s proposal to use cautionary labelling to reduce unhealthy food consumption was subject to trade challenges, notably by the USA, as a violation of the WTO’s Technical Barriers to Trade Agreement (TBT) [10]. These challenges never proceeded to a dispute panel, but Thailand did abandon its ‘traffic-light’ labelling system for a daily guideline label favoured by the food industry.

The trade and food labelling issue recently reappeared in dramatic fashion, following US government efforts during the NAFTA renegotiations to have the new agreement explicitly ban front-of-pack nutrition labelling in the three signing countries (Mexico, the USA, and Canada). Responding to the interests of its processed food industry, the American fear was that Chile’s far-reaching food labelling legislation and regulations (already objected to by the USA) would, with Canada soon to follow suit, spark a global norm cascade similar to that experienced earlier with tobacco warning labels and (now) plain packaging [11]. Industry and those governments opposed to such labelling regulations argue that there is no evidence that they work [12], although the extent of their legal (or trade treaty) efforts to prevent their implementation suggests that they fear the contrary.

Further studies of the trade and nutrition transition are found in other articles in this collection. Cassel’s contribution [13] locates the surge in overweight and obesity rates in the Federated States of Micronesia within a longer history of colonial dependencies (primarily on the USA and Japan), where economic trade helped to create an obesogenic food environment. The shift in these small island nations from a subsistence to a cash economy linked to trade liberalization increased their reliance on imports of inexpensive but nutrient-poor refined foods, the poster-child of which has been frozen turkey tails, deemed unhealthy and inedible in the USA but a ‘food’ commodity worth exporting elsewhere. When Western Samoa joined the WTO in 2012, it was obliged to remove its ban on turkey tail imports that it had imposed for public health reasons and, instead, was encouraged “to develop and implement a nation-wide programme promoting healthier diet and life style choices” [14]. Another study, focusing more on food consumption than on trade itself, and hence not included in this collection, similarly found that in the five Pacific Island countries it studied (Kiribati, Solomon Islands, Vanuatu, Samoa, and Tonga) “on average, imported food was significantly or near significantly associated with both ‘unhealthy’ food and obesity at a population level,” suggestive of the role played by trade. This ecological study, however, also noted variance across the five countries, implying that “the trade-off between trade and healthy diets may not need to be as great as it would seem provided that health sensitive policies are put in place” [15] (p9). Whether trade treaties allow for such policies is a different matter, and one examined in two other studies on the Trans-Pacific Partnership (TPP) agreement described later.

A deeper examination of how trade treaties increase unhealthy dietary changes is provided in the Thow and Hawkes [16] contribution, which focuses on Central America. Similar to the earlier 2006 study, this paper examines three pathways by which diets have been negatively impacted by reductions in tariffs and non-tariff trade barriers under WTO trade rules and those of regional FTAs, such as the 2005 US-Central American Free Trade Agreement (CAFTA) since joined by the Dominican Republic (CAFTA-DR). The study employs trend data to link trade liberalization measures to changes in five categories of US food imports: staple grains and animal feed, meat, dairy, fruits and vegetables, and snacks. Their descriptive analysis finds that trade liberalization is associated with increased availability of most of these foods, and while some traded foods are healthy (e.g. temperate climate imports of grapes and apples), the worrying trend is the region-wide shift from a largely plant-based (and healthy) diet to one with greater quantities of energy-dense and highly processed (unhealthy) food items arising from increased trade with the USA.

### The natural experiments

While highly suggestive, the articles summarized above show robust correlational evidence of the links between trade and investment measures and proximal determinants of poor health (such as those contributing to NCDs and the nutrition transition). But they are just that: highly suggestive but not necessarily causal. One of the strongest research designs for inferring causal relations between trade and health-determining pathways is a natural experiment research design, when comparisons between countries undergoing novel trade or investment treaty measures can be compared with those that are not. The findings of two such natural experiments have appeared in this journal. The first of these examined the impact of Vietnam’s access to the WTO (one of the last countries in the world to do so) and its concurrent investment liberalization agreement with the USA, on sales of sugar-sweetened carbonated beverages (SSCB) [17]. Using rigorous ‘difference in difference’ models, the study compared changes in such sales post-liberalization, with trends over the same time period in a matched control country (the Philippines) with a long history of both liberalized trade and US FDI. As hypothesized, SSCB sales rose significantly in Vietnam while remaining flat in the Philippines, with investment liberalization increasing domestic market dominance by the two US-headquartered transnationals, Coca-Cola and PepsiCo. The study used sales of unprocessed foods as a control commodity, as such foods are unlikely to be targets for FDI, and found little or no change in these healthy foods. Given anticipated (projected) trends, by 2019 increased SSCB consumption in Vietnam will increase per capita sugar intake by almost a kilogram annually, enough to raise health concerns especially when considering other aspects of the nutrition transition that are also underway in that country.

A second natural experiment compared trends in the soft-drink market in two countries: Peru (which had entered a bilateral FTA with the USA) and Bolivia (which had no such agreement) [18]. Using the same study design, this study found little difference in total per capita soft-drink sales volumes between the two countries, but a significant increase in FDI flows into Peru following its FTA with the USA (and no change in FDI in Bolivia), together with a slight (non-significant) decline in SSCB imports into Peru while such imports continued to rise in Bolivia. The implication drawn by the authors is that post-liberalized investment in Peru led to increased domestic SSCB production. Peru’s stagnation in SSCB sales in the country was offset by a notable shift towards other sales in other sugar-sweetened beverages, such as juices and sports/energy drinks. The longer-term health implications of this study are harder to ascertain, and the article concludes with some reflections on challenges in the design and interpretations of findings from natural experiments. Both articles, however, leave little doubt that investment liberalization is now playing a greater role in shaping domestic food and beverage markets than liberalized trade in such commodities.

Less ambiguous are the results of two other natural experiments undertaken by contributors to this collection, albeit reported in articles that were published in other journals. Both studies concerned trade agreements between Canada and the USA. One paper [19] looked at changes in the supply of caloric sweeteners in Canada following tariffs reductions that were part of the 1994 NAFTA agreement. High fructose corn syrups (HFCS), one such sweetener, is primarily produced and used in food and beverage manufacturing in the USA, where half the per capita caloric intake by sweeteners in that country comes from HFCS. Only Canada and Mexico (both NAFTA countries), along with Argentina and Japan, similarly consume HFCS, albeit in much smaller amounts [20]. The study found that tariff reductions on food and beverage syrups containing HFCS were associated with a 41% increase in kilocalorie per capita increase in sweetener supply in Canada. Other matched OECD countries that did not have FTAs with the USA (a design the authors refer to as ‘synthetic controls’) did not experience any such rise. While not claiming causality, the study noted that obesity and diabetes rates in Canada rose over the same study period in parallel with the increase in sweetener supply, and to a greater extent than in other advanced industrialized countries that served as the synthetic controls. A second paper [21] using a similar synthetic control design, but focused on total caloric intake in Canada following its earlier (1989) bilateral trade agreement with the USA, found that US exports and investment into Canada’s food and beverage sector increased in tandem with reductions in trade and investment barriers. Between 1988 and 2006, the years covered by the scheduled reductions, there was an increase in calorie availability in Canada of 170 kcal/capita/day, equivalent to an average weight gain of up to 9.3 kg for men, and 12.2 kg for women. Both studies support other research that finds that FTAs with the USA “create food environments that more closely resemble the unhealthy obesogenic environment that pertains in the U.S” [21] (p641).

### The health impact assessment studies

Given mounting evidence that trade and investment liberalization was creating and globally diffusing new health risks, it is not surprising that public health researchers began focusing on the specific measures in trade and investment treaties that created such risks, primarily but not exclusively through constraining the ‘policy space’ for new public health regulations. Policy space is defined as a country’s freedom to choose the best mix of policies to achieve its health or development goals [22]. The WTO rules of most concern in this regard are those found in the Technical Barriers to Trade (TBT) and the Sanitary and Phytosanitary (SPS) agreements. Both are intended to reduce ‘non-tariff’ barriers to trade by ensuring, under the TBT, that regulations are not more trade-restrictive than absolutely necessary and, under the SPS, that any food or drug safety regulation is supported by an internationally agreed upon standard or is justified by a scientific risk assessment. Specific reference in the SPS is made to standards set by the *Codex Alimentarius*, an international body under the auspices of the WHO and the Food and Agricultural Organization (FAO) but which is frequently criticized for being dominated by food industry scientists [23]. The *Codex* standards are regarded as the minimum level of health or safety below which countries are not supposed to go. When imported into the WTO SPS agreement, however, these standards were flipped and became the ceiling above which countries should not regulate (as this would create a non-tariff trade barrier) unless they had scientific justification. Both agreements reference the WTO General Agreement on Tariffs and Trade (GATT), which allows exceptions for non-discriminatory measures deemed “necessary to protect human, animal or plant life or health”; the ‘necessity test’ that countries must pass, however, is sufficiently stringent that few exceptions have succeeded when challenged by another WTO member [24]. Although flexibilities within trade rules could allow carefully crafted public health regulations to minimize the risk of potential challenge, whether trade rules should place such a burden on health regulators and their governments remains a question of political economy.

This question assumes more centrality in the new generation of FTAs that began proliferating in the 2000s. With negotiations for new liberalization treaties at the WTO largely stalled since the late 1990s, due in large measure to push-back from developing countries, high-income nations such as the USA and the European Union (EU) began negotiating bilateral or regional FTAs as a way of overcoming WTO stagnation. By definition, such FTAs must be WTO-plus (WTO+); that is, they cannot liberalize less than what WTO agreements already permit and so, logically, must contain measures that go beyond those in such agreements. One of the largest FTAs (until the USA under the Trump administration withdrew from it in early 2017) was the Trans-Pacific Partnership (TPP) agreement. Since re-branded the Comprehensive and Progressive TPP (CPTPP), the agreement now brings together 11 countries on both sides of the Pacific Ocean, with new countries seeking to join despite the American departure. Two public health groups (one based in Australia, the other in Canada) undertook health impact assessments (HIAs) of the TPP. Different iterations of the results of these HIAs have appeared in other journals (e.g. see [25–27]), but two of them were published in *Globalization and Health.* The first of these, although technically not a HIA, used media reports and leaked texts of the draft TPP (which, as with most trade agreements, was negotiated under conditions of strict confidentiality) to assess diet-related implications of WTO+ provisions embedded in the new agreement [10]. Although an incomplete picture, owing to the lack of final TPP text, the assessment cautions about potential dietary risks due to WTO+ provisions in the TPP’s TBT, SPS, and intellectual property rights (IPR) chapters, as well as the inclusion of new chapters on government procurement (opening government purchasing contracts to firms based in other TPP countries) and investment protection.

The second article, taking advantage of release of the final TPP text, affirmed many of these early health warnings [23]. In keeping with such FTAs, the IPR chapter, while acknowledging the flexibilities for compulsory generic licensing and parallel importing under the WTO agreement on Trade-Related Intellectual Property Rights (TRIPS), contain many TRIPS+ provisions which would have placed delays in generic competition. As other contributions to this collection point out, this is likely to price many drugs with extended patent protection beyond the affordable reach of most people and governments. Some of these provisions are ‘suspended’ in the CPTPP following US withdrawal, as they had been agreed upon primarily at the insistence of the USA. By interrogating treaty measures clause by clause, this HIA cautioned that the TPP’s SPS+ provisions would weaken use of the precautionary principle (with the WTO SPS rules allowing a modicum of evidence to suffice as scientific justification for regulations exceeding *Codex* standards), and toughens further the ‘necessity test’ under TBT+ provisions, essentially requiring all new health regulations to be fully trade compliant (and necessary) *before* being enacted. Claims that the agreement does not prevent governments “from adopting or maintaining technical rules or standards” (often cited by trade ministers favouring the agreement), this protection is immediately undermined by the *caveat* that such rules or standards must be “in accordance with…obligations under this Agreement” [23] (p3). There are also new obligations requiring governments that are party to the agreement to allow interested individuals (including corporations) from other member countries to participate in regulation setting consultations or meetings, creating the risk of ‘regulatory capture’ by industry interests. The HIA finally itemizes problematic elements in the investment chapter which, although constraining some of the criteria by which foreign investors might sue governments over measures that they believe impinge upon the value of their investment, fail to address the lack of transparency, due process, and conflict of interests still resident in the TPP’s final agreement. The TPP does allow a carve-out from investment rules for any tobacco control measure, leading the HIA to question: why not, then, for all other non-discriminatory public health measures? As these authors point out in a subsequent analysis, the TPP investment rules “lag behind newer reform measures”, with the intergovernmental United Nations Conference on Trade and Development (UNCTAD) now similarly calling for an exclusion from investment rules of all non-discriminatory government legislation and regulations designed to protect health, social, fiscal (taxation), and environmental conditions [28].

Newer generation FTAs are often defended by proponents for their inclusion of chapters on labour and environmental protection, and justified by how such agreements are needed to sustain economic growth. Both defences, as this HIA concludes, are overstated. The TPP labour chapter, for example, only applies to the headline ILO Declaration on labour rights and not to its many specific Conventions; and is enforceable only if a member country lowers its existing labour standards to gain a trade or investment advantage. The environment chapter is similarly cobbled by requiring only that member countries not weaken their existing standards for trade or investment self-interest. While such provisions might slow a trade-related regulatory race to the bottom, they do not incentivize any health protective reach for the top. Whether stronger provisions should be in such treaties remains subject to debate, with some developing countries concerned that this could lead to high-income countries with the resources and capacities to comply with labour and environmental standards using such provisions as a ‘back door’ protectionism against goods from poorer countries.^2^ Economic growth arguments, in turn, rest upon untenable assumptions in conventional econometric modelling (e.g. full employment, equitable income growth, no public costs); even so, various estimates of aggregate economic gain from the TPP show minimal to almost now aggregate gain for most member countries. Some economic sectors win, others lose. When alternative modelling is used that removes the empirically dubious assumptions of conventional (general computable equilibrium) models, the minimal aggregate gains are less, unemployment rises, and income distribution skews towards the top 1%. As the HIA concludes, “Given the paltry economic gains from the TPP, and the various direct and indirect health risks it poses, from a strictly public health vantage, this is not a good Agreement” [23] (p5).

### Access to essential medicines vs. drug patents and profits

Until the recent trade and investment challenges to tobacco plain-packaging laws made by tobacco transnationals (or via supportive governments),^3^ no trade-related issue attracted more public health attention than extended patent protection for drugs, first through the WTO’s TRIPS agreement, and subsequently through FTA TRIPS+ provisions. The broad outlines of this issue are well-known: prior to the WTO TRIPS agreement (considered an outlier for being a ‘protectionist’ rather than liberalizing treaty) many countries had little or no patent protection. TRIPS mandated a 20 year period, putatively to allow drug companies to recover their (usually inflated) costs of new drug discovery [29] (p263). When this was used to prevent generic manufacturing of antiretrovirals (ARVs) during the rapid rise in HIV in South Africa, it sparked a global backlash against the drug companies and led to political and philanthropic initiatives that saw prices on ARVs decline dramatically. In 2001, largely driven by concerns of African countries, the WTO issued its ‘Doha Declaration’ affirming the rights of countries when faced with a public health emergency to unilaterally issue ‘compulsory licenses’ to produce affordable generic drugs. This was later further amended to allow countries lacking domestic pharmaceutical facilities to obtain licenses to import generic drugs manufactured in other nations.

As the first article in this collection argues, such measures, although lauded as ‘watershed moments’ in international trade policy, leave unaddressed the potential for TRIPS+ provisions in new FTAs to undermine the potential gains of these WTO reforms [30]. Some of these TRIPS+ provisions, found in several FTAs, lengthens the patent protection period to compensate for delays in market approvals or grants data exclusivity rights to patent holders, both of which add years of delay to introduction of generic competition. One of the suspended provisions in the TPP’s TRIPS+ chapter would have made it easier for drug companies to continually issue new patents for very minor changes in their formulation or mode of administration, a practice referred to as ‘evergreening’. As this article goes on to point out, the TRIPS reforms in the early 2000s remained silent on underinvestment in drug research on diseases common in low-income countries but rare in wealthier nations. This remains a contentious policy issue globally, with multiple but so far largely unimplemented suggestions for incentivizing research into these ‘neglected diseases’ by delinking the cost of new drug discovery from the eventual market price [31]. Expressly concerned with the rise of TRIPS+ in FTAs, this article cautions that “stark inequalities in power and influence among trading nations” leave “LMICs vulnerable to pressures to permit the globalization of IPRs in order to protect broader trade and economic interests” [30] (p1), an evidence-informed comment that applies to most provisions being negotiated in new FTAs.

A key weakness of TRIPS reforms noted by this article (the unwieldy complexity of so-called Paragraph 6 that allows for parallel importing of generics) is probed in detail in another contribution [32]. This article dives into the political debates surrounding Canada’s efforts to become the world’s first country to pass legislation compliant with Paragraph 6 in order to issue a compulsory licence for a generic drug export to a low-income country. Although the transaction was ultimately successful, developing country perspectives on Paragraph 6 are less than enthusiastic, complaining of its cumbersome requirements while failing to address the need for continued affordable access to essential medicines. This contribution concludes that ‘Canada’s Access to Medicines Regime’ (CAMR) “appears to be more powerful symbolically than in practice” [32] (p8), a prescient comment given that its single parallel importation remains the only one so far attempted globally under Paragraph 6 provisions. The article notes several changes that would be required to make the provisions more effective, including incentivizing generic manufacturers willing to export using Paragraph 6, simplifying the rules under which Paragraph 6 can be actioned, and engaging more broadly with the need to develop pharmaceutical capacities in low-income countries allowing for more frequently invoked compulsory licensing [33]. More recently the challenge of ensuring equitable access to essential medicines for all has led to international policy calls to delink the cost of new drug discovery and production from the eventual price of drugs [31]. Opposition to such efforts continues from some countries with strong patent pharmaceutical interests.

Continuing with a focus on Canada as a case study, one of that country’s most experienced drug researchers examines six instances in which Canada engaged on issues of TRIPS compliance and access to medicines [34]. Updating the previous contribution, Lexchin’s study notes that, with the exception of the Canadian government’s expressed ambivalence when pharmaceutical transnationals attempted to block South Africa’s efforts to access generic ARVs, describing the need to balance between access to medicines and protection of corporate IPRs, in all other instances Canada has prioritized IPRs over access. This stance included twice failing to amend the well-noted shortcomings in its CAMR, and adopting positions in international fora generally supportive of US policies on patent protection in deference to retaining good relations with its major trading partner.

### Broadening the trade/health Nexus

One of the long-standing public health trade concerns is the implication of the WTO General Agreement on Trade in Services (GATS) and GATS+ measures in several subsequent FTAs on access to health services. Trade in health services is driven by commercial considerations, not by whether the growth in health services trade produces equitable outcomes. Given ‘lock-in’ and ‘ratchet’ provisions in many trade agreements, governments that commit to trade in health services may find it difficult to return to public health care services or financing once they have been privatized and opened to foreign competition. At the same time, countries where most health care is provided or financed privately could stand to gain through increased market access in other countries. This possibility is explored in a study of an EU/India trade and investment treaty [35] and provides a useful case study of the different liberalization modes being mooted, and how trade with the EU could positively benefit different health services sectors of the Indian economy. The article also speculates on benefits to the EU itself, in terms of outsourcing certain health system functions to lower cost countries in order to cope with ageing Europeans, increasing health services demands, and long wait lists. These are not new speculations, and have been raised frequently in studies of the health equity impacts of health worker migration or ‘medical tourism’ (what this particular article calls ‘medical value travel’), both of which could reduce access to health services for poor populations in low- or middle-income countries that are losing health workers to migration or catering to privately-paying international patients in hospitals inaccessible to most of the locals [36–39]. The present article, by Indian health economist Rupa Chanda, hints at these concerns, primarily in the discordance between (still largely public) health systems in the EU and India’s extremely privatized systems; and in the perception in most high-income countries that health care is a public good that should be protected from predatory private interests. The article is silent on the human rights implications of international commercial trade in health services, a topic that has raised concerns by several UN Special Rapporteurs on the right to health [40]. It does take a cautious stance, however, arguing for limited experimentation with health services trade; and presages debates over the role of the private sector (in financing, provision, or both) in pursuit of the new WHO (and broader UN Sustainable Development Goal) imperative to achieve universal health coverage, a topic well covered by other contributions to this journal [41–43].

As this journal has made clear in many of its submissions, globalization processes affect health through multiple pathways and not simply through those more directly linked via changes in health systems. The contribution by McNamara is a compelling example of a trade study that attempts to examine the intersection of trade policy reforms on labour market dynamics and social protection policies [44]. Using an innovative methodology (fuzzy-set Qualitative Comparative Analysis, or fsQCA) the study modelled changes in textile and clothing production following the termination of the 2005 Multi-Fibre Agreement (MFA) that rather abruptly ended a prior set of quotas that protected production in some countries while creating import barriers for others. Some low-income countries (e.g. India and Bangladesh) rapidly saw a surge in textile production and export, while others (high-income nations, and earlier outsourcing countries such as Mexico and Romania) experienced rapid declines as the international garment industry pursued lower cost production sites. Using adult female mortality as a health outcome measure (given that most textile workers are women), the lack of access to social protection measures was related to worsening mortality rates in both developing countries (despite rising employment) and developed countries (a result of job-loss). Protective labour regulations and social policies moderated some of the negative effects of employment disruptions arising from the MFA’s demise, but increases in precarious or hazardous employment were also noted as a characteristic outcome, concluding that “social protection may be inaccessible to the type of workers who are vulnerable to processes of liberalization… and that workers can be particularly vulnerable to processes of liberalization due to the structure of their country’s social policies” [44] (p17).

This vulnerability is aggravated by inequities in different countries’ regulatory capacities with respect to FTAs, or what this contribution from Wallis and colleagues [45] calls preferential trade agreements (PTAs). By way of one example, they cite two branches of the US government in 2013 having a combined budget of almost US $500 million and a staff of over 2,000 to ensure that no country violates its pharmaceutical IPRs. The authors argue that such stark scalar differences risks increasing health inequities globally, and that development assistance to poorer countries to assist their trade compliance or readiness (the ‘aid for trade’ rhetoric popular since the dawn of the new Millennium), while possibly useful, can be of far more benefit to high-income donor countries with export or IPR agendas, than to the economic development of aid-recipient nations. On a more positive note, the commentary suggests that the rise in ‘south-south’ collaboration may yield more innovative regulatory solutions to the lack of such capacities that characterize least-developed and most low-income countries.

### Forward research directions

Many of the articles gathered for this collection are based on research findings, incorporating an array of methodologies and methods: structured narrative and scoping reviews, trend and regression analyses, critical assessments of trade policy and trade/investment disputes, text analyses of trade and investment treaties, natural experiments, health impact assessments, qualitative comparative analyses, and key informant interviews. Different methods yield different insights into the trade and investment/health nexus, although establishing causality in the relationship between trade policy, trade and investment liberalization treaties, and specific health outcomes, as with research on most complex social phenomena, remains challenging. Two recent contributions to *Globalization and Health* address head-on challenges facing trade and health researchers in improving the robustness of their findings [46, 47]. Both contributions cited methodological limitations in the current literature, examining studies well beyond those published only in this journal.

The first review, by Barlow and colleagues who include some of their own work, and that published in this journal and discussed above, focused on quantitative study designs. The 17 articles in the review collectively provide consistent evidence on the association between trade agreements and increased consumption of unhealthy commodities (ultra-processed goods, sugar-sweetened beverages) and higher rates of cardiovascular disease incidence, but only inconclusive findings related to tobacco consumption, mortality rates, and life expectancy. Although eleven of the studies were considered to be weak or moderate in methodological strength, six were judged to be of high quality, if also at some risk of bias due to inattention to unobserved confounding mechanisms. Nonetheless, the authors, while recognizing the need for improvements in research design, conclude that the extant evidence suggests that trade agreements do pose significant health risks. They also call for greater interdisciplinary engagement with economics, political science, and psychology to avoid public health/trade researchers becoming too insular; as well as for more detailed study of the specific policies within trade agreements that can account for differences in outcomes, as well as policies that might mediate the trade/health relationship.

The second article, also involving contributors to other contributions to this collection, reaches similar conclusions. Focusing on quantitative studies and review articles examining how trade and investment affects NCDs and NCD risk factors, the contribution notes a number of methodological weaknesses similar to those in the previous article: inconsistencies in examining confounding variables and inadequate testing for endogeneity as well as relying on aggregate rather than sector-specific trade/investment indicators, or failing to separate trade from investment measures. To researchers’ credit, however, only a few studies relied on cross-sectional data with most making use of longitudinal data and sensitivity analyses. The authors conclude that most of the study designs interrogated show moderate methodological strength, noting several ways in which future study strength might be enhanced, including more attention to mediating policies and more specificity in which trade or investment measures conceptually would be likely to influence health outcomes. A particularly interesting finding is that studies strong on conceptual models are weak on empirical evidence, while those generating quantitative analyses tended to be weak on theoretical conceptualization.

### In sum

Although much is being made in the post-Trump era of ‘illiberalism’ of protectionist challenges to the system of multilateral and burgeoning regional trade and investment rules, it is improbable that global trade will disappear any time soon. The specifics of its rules-based content will shift with changes in the (ultimate) politics of who exercises negotiating or autocratic powers over whom, and for whose benefits. The dynamics of international politics and economics, in a context of normative agreements such as the Sustainable Development Goals and the Paris Accord, and with the looming near-present overshoot in many of the world’s ecological systems (climate change being only the most immediately critical one), is certain to generate considerably more critical research and scholarship on the role played by trade policy, trade treaties, and investment agreements on global health now, and in the years to come. We are optimistic that some of this ground-breaking work will continue to appear in the pages (if such is still a reasonable descriptor for on-line journals) of *Globalization and Health*.

## Footnotes

1 National treatment, or non-discrimination, requires countries to treat imported goods no differently than it does their own domestically-produced goods. Most favoured nation means that the best tariffs schedules offered to any other country that is part to the trade treaty must be extended to all other member countries, although some exemptions are allowed for preferential market access for goods from least-developed or low-income developing countries.

2 One example of this is the US demand under a renegotiated NAFTA that a fixed percentage (40–45%) of vehicle content manufactured in Mexico (and integral to the North American automotive supply chain) exported to the USA must be made in factories paying workers at least US $16/h. Although this demand could help to increase Mexican labour rates (with positive health externalities), it could also increase unemployment with manufacturers unable to meet the higher costs except through rapid automation and labour force reductions. Importantly, the rationale for this demand was not to improve livelihoods for Mexicans, but to protect manufacturing for US auto workers [48].

3 The well-known challenges to Australia’s plain-packaging legislation came from two directions. The first, invoking a bilateral investment treaty, involved tobacco transnationals and was led by Philip Morris International, which attempted to sue the Australian government. It was later dismissed on procedural grounds by a tribunal. The second saw five member states of the WTO launch a formal trade dispute (similarly dismissed by a tribunal). Although these challenges were unsuccessful, they did ‘chill’ the plain-packaging norm cascade for several years, with some unsuccessful WTO countries continuing to argue that plain packaging regulations lack any evidence of effect, unfairly discriminate against their tobacco products, and was damaging to their overall economic development [49].
